# Preventing HIV and HSV-2 through knowledge and attitudes: A replication study of a multi-component community-based intervention in Zimbabwe

**DOI:** 10.1371/journal.pone.0226237

**Published:** 2020-01-08

**Authors:** Fang Yu, Nicholas A. Hein, Danstan S. Bagenda

**Affiliations:** 1 Department of Biostatistics, University of Nebraska Medical Center, Omaha, Nebraska, United States of America; 2 Department of Anesthesiology, University of Nebraska Medical Center, Omaha, Nebraska, United States of America; Fordham University, UNITED STATES

## Abstract

**Introduction:**

Approximately two-thirds of HIV-infected individuals reside in sub-Saharan Africa. The region accounts for 68% of the new HIV infections occurring worldwide with almost one-half of these infections being among young adults aged 12–24 years. Cowan and colleagues conducted a community-based, multi-component HIV intervention aimed at youth in rural Zimbabwe. Despite some changes in knowledge and attitudes, the community-based intervention did not affect the prevalence of HIV or HSV-2. We selected this frequently cited study for replication since it incorporates individual-, community-, and structural- level intervention components that are often considered in global HIV/AIDS prevention programs. Additionally, the intervention could be easily scaled-up, which is especially important in the context of limited resources. Although this study indicated no intervention effects in reducing HIV, the authors acknowledged some key methodological challenges. Our replication analysis provided important insights regarding the impact of these challenges to the interpretation of the results of this study.

**Methods:**

Our replication study focused on replicating Cowan’s findings and assessing the robustness of Cowan’s results to alternative analytical models based on their study design. We determined how out-migration occurring during Cowan’s study may have affected the population characteristics, the intervention exposure level, and the study findings. While the original intervention targeted knowledge and attitudes as a mechanism to decrease HIV/HSV-2, the Cowan study evaluated the intervention effects on knowledge, attitudes, and prevalence of HIV or HSV-2 separately. To better identify the pathway describing the interrelationship among the intervention and knowledge, attitudes, and prevalence of HIV or HSV-2, we assessed whether increases in knowledge or attitudes were associated with decreased HIV or HSV-2 prevalence.

**Results:**

We replicated the original findings with minor discrepancies during the pure replication. Our additional analyses revealed that the study population characteristics changed over time in ways that may have affected outcomes. These changes also affected the levels of intervention exposure, with 48.7% males and 75.5% females of the intervention group receiving low-level exposure. Both genders with higher level intervention exposure experienced higher increments in multiple knowledge, attitude, and sexual risk behavior outcomes. Unfortunately, these did not translate to a significant reduction in HIV or HSV-2 regardless of the level and combination of knowledge and attitude domains. However, males receiving high-level intervention exposure compared to control indicated significantly lower odds of having HIV or HSV-2 under a Bayesian modeling paradigm.

**Conclusions:**

Our findings suggest a more robust conclusion on the study intervention effects. Further study based on a design that more consistently maximizes the exposure level of the intervention is necessary and should ideally be an evaluated goal in similar studies. Evaluation of the intervention impact for key subgroups of the target population is important and would better advise the use and scale-up of the evaluated interventions in various contexts. Our observation of a consistent lack of relationship between knowledge/attitudes and HIV/HSV-2 suggests a need to explore and include relevant additional and or complementary interventions, e.g., promoting effective skills in reducing risky sexual behaviors and addressing cultural and structural bottlenecks that may reduce HIV/HSV-2 risk among youth.

## Introduction

Approximately 1.9 million individuals aged 15 years and older become infected with HIV annually [1]. The burden of HIV is especially high in eastern and southern African countries. These countries account for nearly half of the people living with HIV, while home to only 6.2% of the world’s population [1]. Despite some global progress through a comprehensive approach for addressing HIV, new infections of HIV remain quite high in Sub-Saharan Africa, especially among adolescents and young women. It is a public health priority to identify effective HIV prevention interventions among young people in southern Africa [2]. A systematic review performed by the Joint United Nations Program on HIV/AIDS has indicated that school-based interventions can reduce self-reported risky sexual behaviors [3]. Unfortunately, few trials have used biomedical endpoints to evaluate the impact of HIV interventions for young people [4].

In a review of the literature regarding HIV prevention interventions, we evaluated the impact of several papers. We first calculated the citation rate for each paper using the number of citations of the paper from the Web of Science database and months since publication. We then weighted the citation rate with the journal impact factor. A paper by Cowan et al. [2] was one of the top 10 most highly impactful studies in HIV prevention among the most recent 94 studies available in the 3ie Repository.

Importantly, in addition to collecting information related to knowledge, attitude and risk behavior changes, Cowan et al. [2] used biomedical endpoints to evaluate the effectiveness of a rural, multi-component, community-based HIV prevention intervention program for young people. Specifically, the community was defined as the rural health clinics, its catchment population, and the secondary schools. The intervention contained three core components. The components included a youth program for in- and out-of-school youth that focused on self-awareness, gender issues, and HIV prevention; a community-based program for parent and community stakeholders concentrating on reproductive health; and a program for nursing and working staff in rural health clinics that focused on improving clinic accessibility. The study was a cluster-randomized trial conducted in rural Zimbabwe. While the intervention displayed effects in improving some knowledge and attitude outcomes, it reported no impact in reducing risky sexual behavior or HIV or HSV-2 outcomes.

Several interventions, including the Sista2sista and DREAMS programs in Zimbabwe, are being pursued to help curb the HIV/STD epidemic among youth. Some of these interventions focus on increasing knowledge related to HIV risk and also on the promotion of behavioral changes that may alleviate HIV risks at the individual level. At the structural level, there is a question as to whether getting youth into school and keeping them there (especially young girls) will have an impact on HIV/STD risks. Cowan’s study can help clarify whether it is worth pursuing these specific components that address behavioral and some structural aspects versus current comprehensive prevention approaches that may emphasize the acquisition of other risk alleviation skills, e.g., proper condom use. This is especially important given parental, community, and ethical concerns associated with aspects of a broader sexual education approach among African youth [5] as well as limited available resources.

Although Cowan’s study did not demonstrate that the intervention had an effect on HIV and HSV-2 prevalence, there was an improvement in some knowledge and attitude indicators. Given that there were at least 48% of participants with no to low exposure to the intervention in the treatment group, it is unclear whether the null results of the intervention effects on HIV or HSV-2 prevalence were due to insufficient dosage of treatment or lack of treatment efficacy. Also, it is unclear whether no association exists between increased knowledge or attitudes and HIV or HSV-2 prevalence, or whether the effects were too small to be detected.

In the United States, a Community Preventive Services Task Force (CPSTF) [6] recommends comprehensive risk reduction interventions, similar to the intervention used by Cowan. CPSTF suggests that these interventions should be directed to adolescents to promote behaviors that prevent or reduce the risk of pregnancy, HIV, and other sexually transmitted infections. Therefore, the results of this study are relevant globally.

Since the publication of Cowan’s study, the HIV incidence remains high in Zimbabwe. Zimbabwe’s 2017 modes of transmission study [7] shows that the greatest number of new infections, more than 16,000 a year, are occurring among never-married women. Adolescent girls and young women, in particular, experience a dramatically disproportionate burden. Young women (age 20–24 years) had an HIV prevalence that is 2.78 times higher than their male peers. Further, 41% of girls reported that their sexual debut before 18 years was unwanted and rates of transactional sex (non-marital, non-commercial, sexual relationships motivated by an implicit assumption that sex will be exchanged for material support or other benefits [8]) were high and increasing. The DHS 2015 data [9] reflect low and declining knowledge of HIV prevention among young people (46.3% to 41.4% in young women and 41.7% to 41.4% in young men from 2010 and 2015). Thus, adolescents and young people have been prioritized in Zimbabwe’s efforts to revitalize HIV prevention. Initiatives like Sista2sista and DREAMS have been rolled out in hot spot districts to empower young women to make informed sexual reproductive decisions. Program managers, though, note challenges related to low participation in addition to little funding.

We, therefore, selected the frequently cited Cowan study since it incorporates individual-, community- and structural- level intervention components that are often considered for inclusion in global youth HIV/AIDS prevention programs in the context of limited resources. Cowan’s study indicated no intervention effect in reducing HIV or HSV-2 prevalence. However, with our proposed replication methodology applied to the Cowan study, we will be able to provide additional information. This information will be helpful for enhancing the effective use of interventional approaches that integrate otherwise generally accepted and applied key behavioral, biomedical, and structural domain components for prevention activities at the population level [10]. The comprehensive HIV intervention packages, as recommended by WHO consensus, center around biomedical components—including access to HIV testing, prevention, care and treatment for STDs and HIV/AIDs; socio-behavioral components—driven mainly by abstinence, being faithful (reduction of sexual partners) and condom use (i.e. ABC strategy); and structural components–such as cultural, social, policy factors including gender empowerment, poverty, discrimination, and stigmatization. Underlying socio-behavioral components is the dogma that providing youth with information or increasing their knowledge regarding HIV risk or safety will drive change in key behaviors associated with these socio-behavioral components.

Furthermore, the authors noted methodological challenges that may have affected their findings. These challenges are critical to enhancing our understanding of how to effectively implement interventions measured at the population level using biomedical indicators. Our replication approach puts greater consideration on addressing these challenges. Specifically, our replication study will examine the potential impact of these challenges on interpreting the study results. The interpretation is critical in guiding current public health practice and policy development, as well as methodological approaches for addressing similar challenges common in other studies. Additionally, for policy advising purposes, it is essential to understand the extent to which the above-mentioned core components of the intervention used in Cowan’s study can be varied and still achieve the desired effect. For example, we believe that if youth with suboptimal exposure levels of the envisaged key components of the intervention can be shown to still experience a significant reduction in the key outcomes, then perhaps, for some subgroups of youth, the outcomes could possibly be robust to some variation during scale-up.

In pursuit of these goals, we conducted an independent replication study through a push-button replication (PBR), a pure replication, measurements and estimation analyses (MEA), and a theory of change (TOC) analysis [11, 12] to assess the robustness of the original findings. All these analyses were conducted in line with our published replication plan [13]. We believe that our replication study will help us (i) verify the findings in Cowan’s study [2]; (ii) improve our knowledge and ability to provide practical guidance of the utility of community-based knowledge, attitude, and behavioral change interventions on preventing HIV, STDs, and pregnancy among African youth within the context of a comprehensive approach; (iii) provide analytical guidance addressing methodological challenges that affect the level of intervention exposure across participants; and (iv) guide policy toward more targeted and efficient programs and studies with potential for affecting HIV prevalence in both African and US communities.

## Methods and materials

### Background

Cowan’s study [2] was conducted among young adults aged 18–22 years in South-Eastern Zimbabwe. A total of 30 communities were randomized to early intervention (implemented in 2003) or delayed implementation (implemented in 2007). The intervention aimed to achieve change in societal norms within communities. It was comprised of three integrated components: 1) the youth program for in- and out-of-school youth—intended to enhance knowledge and develop skills; 2) a 22-session community-based program for parents and community stakeholders, which aimed to improve knowledge about reproductive health, communication between parents and their children, and community support for adolescent reproductive health; 3) a training program for nurses and other staff working in rural clinics, which aimed to improve accessibility of clinics for young people. The impact of the intervention was assessed four years later, using both self-completed paper surveys and audio computer-assisted surveys [2]. Briefly, the attitude and knowledge variables were measured based on questions in 11 domains. All questions were graded as “correct” or “incorrect” depending on whether the participant agreed or disagreed with the statement. Each domain was summarized using binary variables depending on whether participants answered all relevant questions correctly, or answered seven or more out of 10 questions correctly regarding attitudes toward control over sex, or answered four or more out of eight questions correctly regarding attitudes towards the Jewkes scales. The Jewkes scale is the adapted Gender Equitable Men (GEM) scale [14] suggested by Rachel Jewkes (Director, Gender & Health Research Unit, South African Medical Research Council). Blood samples and urine samples were collected at baseline in 2003, interim evaluation time in 2006, and final evaluation in 2007. The blood samples were used to test for HIV and HSV-2 antibodies, and a urine pregnancy test was also conducted for young women. The primary endpoints of the trial were the prevalence of HIV and HSV-2. The secondary endpoints were knowledge and attitudes related to HIV or STD prevention; sexual behavior and reproductive health; clinic attendance; and pregnancy prevalence. The details on the definitions of all primary and secondary variables can be found in Table 1.

**Table 1:** Variables analyzed by both original paper and the replication study.

Variables	Outcome (# of survey items) or categories
**Table 1 in Cowan [2]: Characteristics of participants (containing missing values except for age)**	
Age	18 years; 19–20 yrs; 21–22 yrs
Religion	Catholic; Anglican; Apostolic; Pentecostal; Other/none
Ever married	Yes; No
Married aged ≤ 16 years	Yes; No
Lived in community ≥ 5 years	Yes; No
Level of education	None/primary only; F1-2; F3-4; F5 or higher
Orphan status	Non-orphan; Lost one/both parents
Socio-economic status (multiple variables)	Cannot afford soap to wash clothes; Child/children in house receiving external assistance; Adult in house skipped meal in last week; Participant gone day without food in last week; (All are binary: Yes/No)
Attended Regai Dzive Shiri (RDS) study school	Control school; Intervention school; Non-RDS school; No secondary education;
**Tables 2a & 2b in Cowan [2] (Part 1): Knowledge and attitudes outcomes (responding correctly to all questions if not specified: Yes/No)**	
Knowledge and self-efficacy	HIV acquisition (3)*; STD acquisition (2)*; Pregnancy prevention (2)*; Condom self-efficacy (3); Sexual refusal self-efficacy (2); HIV-testing self-efficacy (3)
Attitudes—Control over sex	All responses correct (10); ≥ 7/10 questions responded correctly" (10)*; Control around sexual refusal (3)*; Control around sexual partners (4)*; Safe sex and condoms (2)*
Attitudes—Jewkes scale: Gender empowerment	≥ 4/8 responses "correct" (8)*; Right to refuse sex (2)*; Rights within marriage (2)
**Tables 2a & 2b in Cowan [2] (Part 2): Behavior outcomes (Yes/ No)**	
Control over life & future	Have long-range goals (1)
Reported sexual behavior	Ever had sex; Sexual debut 17 or younger; Two or more lifetime partners; Two or more partners in last 12m; Did not use condom last sex
Reported pregnancy prevention	No pregnancy prevention used with first partner; No pregnancy prevention used with last partner; No pregnancy prevention used with any partner
Clinic attendance and perception of staff	Been to clinic in last 12 months; Never worry that clinic staff will tell others purpose of my visit; Always seen in private, never worry that other patients will know the purpose of my visit; Would go to clinic for treatment if had discharge from penis (males only); Able to go to clinic if I needed to get contraception (females only)
**Table 3a & 3b in Cowan [2]: biological outcomes (Yes/ No)**	
Reported symptoms of STDs	Ever had symptoms of STD; Sought treatment for STD symptoms; Genital discharge prevalence; Genital warts or sores prevalence; Prevalence of any symptoms of STD
Primary biological outcomes	HIV infection*; HSV-2 infection*
Pregnancy and reported pregnancy (females only, analyzed by all, unmarried and married women, separately)	Currently pregnant; Reported unwanted pregnancy; Reported past or current pregnancy; Reported aborted pregnancy; Any evidence of pregnancy*;

*Also analyzed in the sub-analysis restricted to survey participants who attended a Regai Dzive Shiri trial school and had lived in the community for the duration of the intervention.

The original intention was to determine the impact of the intervention within a cohort of Form 2 pupils (9th school year) attending trial secondary schools. During an interim analysis conducted in 12 of the 30 communities in 2006, approximately 46% of the original cohort was lost to follow-up due to out-migration, and those who remained were of lower HIV risk than those who left. Specifically, the estimated HIV prevalence was 1.2% (95% CI = 0.7–1.9%) for the remaining cohort, while the community HIV prevalence was 8.3% (95% CI = 7–9.8%) for females and 3.3% (95% CI = 2.6–4.1%) for males. There was also a drop in secondary school attendance in Zimbabwe as a result of the political and economic challenges facing the country, so intervention activities shifted from schools to communities. To optimize the power of detecting a difference in HIV prevalence, the investigators modified the study design to a cross-sectional population-based survey in 2007. Specifically, six enumeration areas from each community were selected. Individuals 18–22 years old within the selected areas were recruited to participate in the survey. The authors noted that no more than 7% of participants of the baseline survey participated in the final survey.

Cowan’s study conducted separate intent-to-treat analyses for males and females. The heterogeneity of sociodemographic characteristics of the final evaluation survey participants was compared between study arms. The unadjusted odds ratios (UOR) and adjusted odds ratio (AOR) were computed using generalized estimating equations (GEEs) with age, strata used for randomization, marital status, and education as fixed effects and exchangeable correlation and robust standard errors, which allowed for intra-class correlation among clusters.

There were 4,684 participants, of whom 55.5% were female in 2007 [2]. Both men and women from the intervention communities had a moderate improvement in knowledge and attitudes. Specifically, the participants in the intervention group were more likely to provide correct responses to knowledge questions. The AOR for questions related to STD was 1.59 (95% CI = (1.27–1.99)) for males and 1.45 (95% CI = (1.17–1.79)) for females. For questions related to pregnancy prevention, the AOR was 1.59 (95% CI = (1.27–1.99)) for males, and 1.32 (95% CI = (1.14–1.55)) for females. In addition, males in the intervention group were more likely to correctly answer attitude questions regarding “control around sexual refusal” (AOR = 1.22, 95% CI = 1.02–1.47)), and “rights within marriage” (AOR = 1.79; 95% CI = (1.05–3.04)). The female intervention group had higher odds of correctly answering all questions on “condom self-efficacy” (AOR = 1.22; 95% CI = (1.01–1.48)), “HIV-testing self-efficacy” (AOR = 1.22; 95% CI = (1.03–1.44)), and attitudes toward “safe sex and condoms” (AOR = 1.24; 95% CI = (1.03–1.48)). The female intervention group was also associated with larger odds of having “≥ 7/10 questions responded correctly” regarding attitudes toward control over sex (AOR = 1.34; 95% CI = (1.11–1.63)), “≥4/8 responses correct” related to gender empowerment (AOR = 1.32; 95% CI = (1.05–1.66)), and that she was “able to go to clinic if she needed to get contraception” (AOR = 1.33; (95% CI = (1.05–1.69)). Females in the intervention communities were less likely to report ever having been pregnant (AOR = 0.64; 95% CI = (0.49–0.83)), while there was no impact on current pregnancy. However, there was no intervention impact on reducing prevalence of HIV (AOR = 1.20; 95% CI = (0.66–2.18) for males, AOR = 1.52; 95% CI = (0.98–2.37) for females) or prevalence of HSV-2 (AOR = 1.23; 95% CI = (0.69–2.18) for males, AOR = 1.23; 95% CI = (0.91–1.66) for females).

### Methods of PBR, and pure replication

In the PBR, we used the code provided by the original authors to reproduce the published results. In the pure replication, using the methods presented in the paper, we reconstructed the variables needed for analyses and performed the analyses described in the original manuscript. In both the PBR and the pure replication, discrepancies were defined as a difference of five-hundredths of a unit or more for odds-ratios and confidence intervals or differences of a percent or more for proportions. Upon occurrences of discrepancies between the pure replication and original results, we used the STATA do-files provided by the authors to identify the source of these discrepancies.

### Methods of MEA

#### MEA for robustness check of alternative modeling

We conducted MEA to examine the robustness of the original results using two alternative modeling strategies based on the study design. For our first robustness check, we applied Generalized Linear Mixed Models (GLMM) to account for the hierarchical structure of the data. Cowan et al. [2] used GEE to account for correlation within communities with robust standard errors. GEE models with less than 40 clusters can underestimate variance, leading to inflated type I errors [15]. GLMM models are less biased when clusters and intra-class correlation is small [16]. In keeping with the original paper, we included age, strata, marital status, and education as fixed effects, and community as a random effect to account for correlation within communities, and robust standard errors were calculated. The same rule used in the PBR and pure replication was used for detecting discrepancies in the odds ratios and their confidence intervals.

Secondly, we evaluated the impact of the intervention on multi-level attitude or knowledge outcomes using ordinal/multinomial models. The knowledge and attitudes of the participants were collected using multi-item survey questions. Cowan et al. [2] categorized the knowledge and attitudes into binary variables using the median as the cutoff value. Instead, we categorized knowledge and attitudes outcome data into quartiles to maintain additional information. An ordinal logistic regression was fit if the proportional odds assumption was met, and a multinomial model was fit otherwise. The proportional odds assumption was tested using the Brant test [17]. Robust standard errors allowing for intragroup correlation among communities were calculated. Since these analyses considered multi-level outcomes instead of binary outcomes as in the original study, discrepancies were claimed to occur if statistical significance and/or direction of point estimate of the odds ratios changed.

#### MEA for assessing the influence of migration

Cowan et al. [2] adjusted their analytical plan due to a substantial migration of participants detected during an interim analysis. Migration may affect the study participants’ demographic characteristics and their exposure to the intervention. Therefore, we performed an additional MEA following a pre-specified analytical plan to evaluate the changes in demographics during the study, the received intervention exposure levels, and their influences on the original results.

First, we examined the representativeness of the final survey participants by comparing characteristics of participants who had been in the community for the entire intervention versus those who migrated to the community during the intervention (newcomers). Chi-Square test or Fisher’s exact test was used to compare the newcomers and individuals who have lived in the community for five years.

Either due to the migration of participants or some other latent cause(s), approximately 50% of participants in the intervention group had no or limited exposure to the intervention according to Supplementary Figure 2 in Cowan et al. [2]. To account for this lack of exposure, we evaluated the impact of the intervention based on the level of exposure the participants received using the GEE approach. We split the intervention group into three distinct groups based on their actual intervention exposure levels. The limited intervention group contained participants with no/limited actual exposure to the intervention or the participants who attended a trial school with peer educators, but lived in the community for less than five years or attended a trial school without peer educators. The moderate intervention group contained participants who either lived in the community for more than five years and attended a trial school with peer educators, or attended 10 or more out-of-school youth (OOSY) sessions, but not both. The high intervention group contained participants who lived in the community for more than five years, attended both a trial school with peer educators and 10 or more OOSY sessions. We evaluated the impact of different levels of exposure to the intervention on HIV prevalence and other key outcome variables using a GEE model adjusting for age, strata, marital status, and education per the original analysis. When GEE failed to provide valid estimates (i.e., separation issues were encountered), an alternative Bayesian logistic regression model with random community effects was fit. These random community effects were included to account for possible unobserved heterogeneity occurring among participants staying in the same community, which is similar to the correlation structure used in the original analysis. We set non-informative priors (i.e., normal distribution with 0 for the mean and 1000 for the variance for the prior distribution of the coefficients, and inverse-Gamma distribution with a mean of 1 and variance of 1000 for the variance parameters of the random effects). The model was fit using the STATA command BAYESMH with a burn-in of 5000 iterations and 50,000 MCMC iterations. The Bayesian approach with appropriate non-informative priors enabled us to produce valid parameter estimates close to traditional maximum likelihood estimates [18].

As an alternative to the dose-response model, we also examined the effect of treatment on the treated using the instrumental variable (IV) approach. A bivariate probit model was used to fit the model with robust standard errors clustered by community. Goodness of fit was established using Murphy’s score test [19]. Additionally, the correlation between errors was examined and reported. If the correlation between error terms was not different from zero, a single probit model was deemed appropriate.

#### MEA for identifying patient subgroups with heterogeneous intervention effects

We anticipated that the association between the intervention and HIV or HSV-2 prevalence would be different for participants of different ages, according to Supplementary Figure 3 in Cowan et al. [2]. Therefore, we evaluated the heterogeneous impacts of the intervention on HIV or HSV-2 among different age or history of risky sexual behavior groups using GEE models. We defined the past sexual history as no risk, low risk, or high risk based on their reported history of sexual behavior. Specifically, the participants with no sexual behavior risk were the participants who reported to have no past sexual behavior. The participants with low sexual behavior risk were the participants who reported to have sexual behavior but no early sexual debut (< = 17 years old), no multiple partners, and reported condom use in the past 12 months. The rest of the participants with valid data reported on sexual behavior history were considered to have a sexual behavior of high risk. The interactions between age or history of sexual behavior and intervention exposure were tested. If there was a significant interaction, a stratified analysis by age or by risky sexual behavior history was performed.

### Methods of TOC analyses on the association between knowledge/attitudes and HIV or HSV-2 prevalence

There may be a potential interrelation among the intervention, knowledge and attitudes, and HIV or HSV-2 prevalence as well as associated sexual risk behavior. Cowan et al. [2] examined the intervention impacts on knowledge, attitudes, HIV prevalence, or HSV-2 prevalence separately. Since the intervention group contained participants with different exposure levels to the intervention, it remains unclear whether the potential null effects of the intervention on HIV or HSV-2 prevalence were due to selection bias, or how the knowledge and attitudes of the participants directly impacted HIV or HSV-2 prevalence. Therefore, we evaluated the effects of knowledge and attitudes on HIV or HSV-2 prevalence via two different TOC analyses.

To account for the influence of the intervention on knowledge and attitudes, we considered the intervention as an IV and evaluated the effects of knowledge or attitudes on HIV or HSV-2 prevalence. The knowledge and attitude variables were quantified using the total number of correct answers in the corresponding survey domain [2]. Probit models with continuous endogenous regressors (ivprobit) were fit with age, marital status, education, and strata as fixed effects. Robust standard errors, clustered by communities were calculated using maximum likelihood. If the relationship between the IV and endogenous variable was not strong, then the IV estimates were biased [20]. Endogeneity was tested using the Wald test for exogeneity. Additionally, the instrument (randomization to intervention) was considered to have a strong relationship with the endogenous variable (knowledge or attitude) if the F-statistic was greater than 10. If these assumptions were not met, a GEE was used with knowledge or attitude included as a fixed effect.

We also examined whether an overall improvement in knowledge and attitude was associated with a reduction in HIV prevalence or HSV-2 prevalence. The knowledge and attitude domains were combined using factor analysis with polychoric correlations [21, 22]. Regression scoring was used to create a new variable that represented overall knowledge and attitudes. This new variable was used in place of knowledge or attitude domains in the aforementioned TOC analysis.

All analyses were stratified by sex as the original paper. The analyses were conducted using STATA version 14.1 (StataCorp, College Station, Texas). The original authors used STATA 10. We anticipated that using a more recent version of STATA did not have an impact on the results. The STATA procedures utilized for this analysis included TABULATE, XTGEE, MEGLM, OLOGIT, MLOGIT, BAYESMH, IVPROBIT, POLYCHORIC, and FACTORMAT.

## Results

### PBR and pure replication

To aid the reader, we have supplied Table 1, which lists the original tables of the manuscript along with the outcomes being analyzed. The PBR and the pure replication both managed to reproduce the original results with some discrepancies. Table 2 summarizes the discrepancies between our pure replication results and the original results. Additionally, the complete PBR and pure replication results can be found in our replication paper [23].

**Table 2:** Discrepancies between the replication analyses and the original results.

Tables in Cowen [2]	Variables	Pure Replication vs Original	MEA using GLMM vs Original	Comments
1: Characteristics of participants	"married aged ≤ 16 years"	Females: Lower proportions	NA*	Variables coded differently
	"lived in community ≥ 5 years"	Lower proportions for both genders	NA	Variables coded differently
2a (males) & 2b (females): Impact of intervention on knowledge, attitudes, and behaviors.	“HIV/STD acquisition”	Higher proportions for both genders	NA	Variables coded differently
	“pregnancy prevention”	Higher proportions for both genders	NA	Variable coded differently
	responding "never worry that clinic staff will tell others the purpose of my visit"	Lower proportions for both genders; Males: AOR switched directions. AOR = (0.76; 95% CI = (0.44–1.30)) vs original: AOR = (1.10; 95% CI = (0.81–1.51)); Females: Higher AOR of 1.16 (95% CI = 0.65–2.05) vs original: AOR = (1.04; 95% CI = (0.80–1.36))	Similar to pure replication results. Males: AOR = (0.76; 95% CI = (0.44–1.29)); Females: AOR = (1.14; 95% CI = (0.65–1.99))	Variable coded differently
3a (males) & 3b (females): Impact of intervention on biological outcomes	HIV infection	Males: Higher proportion of control participants with HIV (7.3% vs original: 7.2%); Males: Narrower CI for AOR AOR = (1.16; 95% CI = (0.64–2.10)) vs Original: AOR = (1.20; 95% CI = (0.66–2.18))	Males: Wider CI for AOR: AOR = (1.22; 95% CI = (0.65–2.29)) vs Original: AOR = (1.20; 95% CI = (0.66–2.18))	Variable coded differently
	HSV infection	Similar to original results.	Males: Smaller AOR of 1.18 (95% CI = 0.68–2.03) vs original: 1.23 (95% CI = (0.69–2.18))	Variable coded differently
	married women reported aborted pregnancy	Higher AOR of 1.31 (95% CI = 0.75–2.31) vs original: AOR = (1.20; 95% CI = (0.63–2.26))	NA	Restricted to married women who reported on education
	unmarried women reported aborted pregnancy	Wider CI for AOR: AOR = (1.00; 95% CI = (0.43–2.33)) vs original: AOR = (0.98; 95% CI = (0.42–2.25))	NA	Education was re-categorized for reaching convergence
4: Sub-analysis of those attended RDS school and lived in community for duration of intervention		Row totals, proportions, UOR and AOR	NA	Analyses based on “lived in community ≥5 years” that differed from original results

* NA indicates that this measure was not replicated in the MEA.

For the PBR, we did not reproduce the numbers and the percentages of participants “Lived in community ≥ 5 years”. Additionally, we do not have the original code to reproduce results on “reported pregnancy prevention” for both genders, *“*would go to clinic for treatment if had discharge from penis” for males, and “able to go to the clinic if I needed to get contraception” for females.

For the pure replication, results were consistent with the original results except in one instance (Table 2). In Table 2A (Impact of intervention on knowledge and attitudes for males) of the original study, the point estimate of the odds ratio of “never worry that clinic staff will tell others purpose of my visit” switched directions but stayed statistically non-significant. This discrepancy was a result of how the outcome was coded. This variable was defined based on the survey question “when I visit my local clinic, I will be treated confidentially.” When the participants reported “always” to the question, they indicated that the staff will not leak their information. If the participants reported “never” or “sometimes” to the question, the participants thought that the staff did not treat them confidentially. Therefore, we interpreted “always” to the original survey question as the correct response for defining the variable “never worry that staff will tell others purpose of my visit,” while the original authors treated “never” as the correct response.

The remaining discrepancies of the pure replication resulted from how missing values were coded. The original authors used inconsistent coding schemes for missing data. For three categories, “Knowledge and self-efficacy,” “Attitudes–control over sex” and “Attitudes–Jewkes scale,” they classified a variable as missing if any item associated with the variable was missing. For “HIV acquisition,” “STD acquisition,” and “Pregnancy prevention,” they coded these variables with missing data as zero unless all questions associated with a variable were missing. In our replication study, we classified a variable as missing if any item associated with it was missing for all aforementioned variables. These different coding schemes resulted in slightly different sample sizes but did not change the number of participants responding correctly to questions or the corresponding UOR, and AOR. The pure replication confirmed improvements in knowledge and attitudes among individuals in the intervention communities and values did not change in significance. Nevertheless, the pure replication also found that the intervention did not have an impact on self-reported sexual behavior, HIV prevalence, or HSV-2 prevalence.

### Results of MEA

#### MEA for robustness check of alternative modeling

Our first robustness check considered GLMM as opposed to a GEE for the data analyses. These analyses indicated similar results to our pure replication, as shown in Table 2.

For our second robustness check, we categorized the outcomes using quantiles as opposed to dichotomizing the outcome. The proportional odds assumption was met for all models except the model on knowledge outcome among females. Therefore, a multinomial logistic model with the first quartile as the reference category was utilized for modeling knowledge among females, and the ordinal logistic regression was utilized in all other analyses. These analyses resulted in increased power in assessing intervention effects on some of the knowledge and attitudes outcomes. Specifically, the intervention effects on the odds of reporting higher level “control over sex” for both males and females (AOR = (1.20; 95% CI = 1.00–1.43) for males; AOR = (1.32; 95% CI = 1.10–1.58) for females) and the intervention effects on the odds of reporting higher level “attitudes toward Jewkes scales” (AOR = 1.27; 95% CI = 1.07–1.51) for males became statistically significant when modeled using ordinal regression. A summary of the discrepancies and detailed results from this robustness check can be found in S1 Table.

The MEA for robustness check to alternative modeling confirmed the original authors’ main conclusions.

#### MEA for assessing the influence of migration

Tables 3 and 4 revealed that newcomers and longtime residents differed in five distinct characteristics. More male and female newcomers belonged to older age categories than participants who lived in the community for at least five years. 35% (control) and 36% (intervention) of male newcomers were 21–22 years old versus 26% (control) and 30% (intervention) of male individuals who had lived in the community for five or more years. Similarly, for females, 35% (control) and 39% (intervention) of newcomers were 21–22 years old versus 27% (control) and 32% (intervention) of female individuals who had lived in the community for five or more years. Secondly, a higher proportion of newcomers than longtime residents completed F5 or above, which is equivalent to high school or higher in the USA. Thirdly, in the intervention group, the newcomers had a much smaller proportion among them attending an intervention school compared to longtime residents. 39% (intervention) of male newcomers participated in a Regai Dzive Shiri (RDS) school while 71.7% (intervention) of longtime male residents attended an RDS-school. Similarly, 26% (intervention) of female newcomers attended an RDS-school in comparison to 61.7% (intervention) of longtime female residents. Fourthly, for both the intervention and control groups, there was a significantly higher proportion of married females among the newcomers compared to longtime residents. Lastly, smaller proportions of male new comers than longtime male residents reported some economic difficulties (i.e. children in house receiving external financial, food, or education assistance in the control group; “can not afford soap to wash clothes”, and “experience a day without food in the past week” in the intervention group).

**Table 3:** Participants’ demographic characteristics stratified by time in community and gender^2^.

	Male n (%)						Female n (%)					
Characteristic	<5 years	≥5 years		<5 years	≥5 years		<5 years	≥5 years		<5 years	≥5 years	
	Control (n = 246)	Control (n = 661)	P-value^1^	Intervention (n = 284)	Intervention (n = 713)	P-value^1^	Control (n = 498)	Control (n = 698)	P-value^1^	Intervention (n = 479)	Intervention (n = 621)	P-value^1^
**Age:**												
18 years	71 (28.9)	259 (39.2)		80 (28.2)	272 (38.2)		162 (32.5)	297 (42.6)		141 (29.4)	250 (40.3)	
19–20 years	90 (36.6)	233 (35.3)		101 (35.6)	225 (31.6)		161 (32.1)	212 (30.4)		152 (31.7)	175 (28.2)	
21–22 years	85 (34.6)	169 (25.6)	0.005	103 (36.3)	216 (30.3)	0.011	175 (35.1)	189 (27.1)	<0.001	186 (38.8)	196 (31.6)	<0.001
**Religion:**												
Catholic	59 (24.0)	121 (18.3)		47 (16.6)	149 (20.9)		84 (16.9)	133 (19.1)		81 (16.9)	133 (21.4)	
Anglican	61 (24.8)	190 (28.7)		82 (28.9)	173 (24.3)		124 (24.9)	172 (24.6)		114 (23.8)	167 (26.9)	
Apostolic	48 (19.5)	132 (20.0)		51 (18.0)	144 (20.2)		111 (22.3)	172 (24.6)		110 (23.0)	113 (18.2)	
Pentecostal	22 (8.9)	61 (9.2)		31 (10.9)	58 (8.1)		76 (15.3)	78 (11.2)		69 (14.4)	64 (10.3)	
Other/None	49 (19.9)	152 (23.0)		70 (24.7)	187 (26.2)		100 (20.1)	135 (19.3)		102 (21.3)	140 (22.5)	
Missing	7 (2.9)	5 (0.8)	0.072^†^	3 (1.1)	2 (0.3)	0.120^†^	3 (0.6)	8 (1.2)	0.295^†^	3 (0.6)	4 (0.6)	0.052^†^
**Ever married**	19 (7.7)	42 (6.4)		25 (8.8)	50 (7.0)		254 (51.0)	264 (37.8)		255 (53.2)	240 (38.7)	
Missing	1 (0.4)	5 (0.8)	0.679^†^	3 (1.1)	1 (0.1)	0.075^†^	2 (0.4)	1 (0.1)	<0.001^†^	1 (0.2)	2 (0.3)	<0.001^†^
**Married aged ≤16Yrs**	1 (5.3)	0 (0.0)		0 (0.0)	0 (0.0)		31 (12.1)	52 (19.6)		35 (13.7)	46 (18.9)	
Missing	7 (36.8)	13 (30.2)	0.270^†^	8 (32.0)	14 (27.5)	0.789^†^	48 (18.7)	42 (15.8)	0.059^†^	35 (13.7)	37 (15.2)	0.219^†^
**Level of education:**												
None/Primary only	26 (10.6)	72 (10.9)		32 (11.3)	75 (10.5)		80 (16.1)	86 (12.3)		62 (12.9)	93 (15.0)	
F1–2	30 (12.2)	73 (11.0)		40 (14.1)	85 (11.9)		63 (12.7)	96 (13.8)		72 (15.0)	93 (15.0)	
F3–4	129 (52.4)	446 (67.5)		156 (54.9)	461 (64.7)		288 (57.8)	456 (65.3)		281 (58.7)	385 (62.0)	
F5 or higher	59 (24.0)	68 (10.3)		56 (19.7)	88 (12.3)		64 (12.9)	57 (8.2)		63 (13.2)	49 (7.9)	
Missing	2 (0.8)	2 (0.3)	<0.001^†^	0 (0.0)	4 (0.6)	0.011^†^	3 (0.6)	3 (0.4)	0.013^†^	1 (0.2)	1 (0.2)	0.050^†^
**Orphan status:**												
Non-orphan	115 (46.8)	336 (50.8)		139 (48.9)	391 (54.8)		253 (50.8)	388 (55.6)		259 (54.1)	340 (54.8)	
Lost one/both parents	129 (52.4)	320 (48.4)		141 (49.7)	313 (43.9)		242 (48.6)	306 (43.8)		215 (44.9)	277 (44.6)	
Missing	2 (0.8)	5 (0.8)	0.516^†^	4 (1.4)	9 (1.3)	0.220^†^	3 (0.6)	4 (0.6)	0.239^†^	5 (1.0)	4 (0.6)	0.755^†^

Notes

^1^Two-sided Chi-Square test of independence or Fisher exact test (noted by^†^).

^2^These analyses that compare the newcomers vs those lived over 5 years, were not conducted by the original study

**Table 4:** Participants’ socioeconomic status and RDS school attendance stratified by time in community and gender^3^.

	Male n (%)						Female n (%)					
Characteristic	<5 years	≥5 years		<5 years	≥5 years		<5 years	≥5 years		<5 years	≥5 years	
	Control (n = 246)	Control (n = 661)	P-value^1^	Intervention (n = 284)	Intervention (n = 713)	P-value^1^	Control (n = 498)	Control (n = 698)	P-value^1^	Intervention (n = 479)	Intervention (n = 621)	P-value^1^
**Socioeconomic status:**												
Cannot afford soap to wash clothes	41 (16.7)	149 (22.5)		50 (17.6)	177 (24.8)		95 (19.1)	148 (21.2)		96 (20.0)	135 (21.7)	
Missing	14 (5.7)	26 (3.9)	0.101	21 (7.4)	36 (5.1)	0.024	22 (4.4)	19 (2.7)	0.212	21 (4.4)	26 (4.2)	0.801
Child/Children in house receiving external assistance^2^	33 (13.4)	128 (19.4)		68 (23.9)	148 (20.8)		86 (17.3)	107 (15.3)		74 (15.5)	95 (15.3)	
Missing	3 (1.2)	3 (0.5)	0.040	1 (0.4)	6 (0.8)	0.441	3 (0.6)	2 (0.3)	0.412	0 (0.0)	4 (0.6)	0.268
Adult in house skipped meal in last week	40 (16.3)	105 (15.9)		44 (15.5)	133 (18.7)		85 (17.1)	141 (20.2)		75 (15.7)	117 (18.8)	
Missing	3 (1.2)	4 (0.6)	0.581	1 (0.4)	2 (0.3)	0.446	4 (0.8)	3 (0.4)	0.286	2 (0.4)	1 (0.2)	0.276
Participant gone day without food in last week	38 (15.5)	92 (13.9)		29 (10.2)	122 (17.1)		65 (13.1)	110 (15.8)		63 (13.2)	87 (14.0)	
Missing	3 (1.2)	3 (0.5)	0.315	1 (0.4)	3 (0.4)	0.016	2 (0.4)	1 (0.1)	0.257	2 (0.4)	2 (0.3)	0.899
**Attended RDS study school:**												
Control school	87 (35.4)	480 (72.6)		11 (3.9)	11 (1.5)		140 (28.1)	480 (68.8)		21 (4.4)	9 (1.5)	
Intervention school	12 (4.9)	9 (1.4)		110 (38.7)	511 (71.7)		28 (5.6)	11 (1.6)		124 (25.9)	383 (61.7)	
Non-RDS school	109 (44.3)	90 (13.6)		112 (39.4)	108 (15.2)		229 (46.0)	87 (12.5)		252 (52.6)	120 (19.3)	
No secondary education	32 (13.0)	71 (10.7)		43 (15.1)	73 (10.2)		91 (18.3)	106 (15.2)		70 (14.6)	104 (16.8)	
Missing	6 (2.4)	11 (1.7)	<0.001	8 (2.8)	10 (1.4)	<0.001	10 (2.0)	14 (2.0)	<0.001	12 (2.5)	5 (0.8)	<0.001

Notes

^1^Fisher exact test used.

^2^External assistance includes financial, food, and/or education assistance provided by government or aid.

^3^These analyses that compare the newcomers vs those who lived over 5 years, were not conducted by the original study.

Table 3 and Table 4 confirm that newcomers and longtime residents have different characteristics. These different characteristics and/or some unknown latent variable may be the cause of different exposures to the intervention that participants received. Specifically, a small number of participants were categorized as being highly-exposed to the intervention. Among females, only 27 (2.2%) had high levels of exposure to the intervention, 273 (22.3%) received moderate levels of exposure, and 925 (75.5%) received no or low levels of exposure. Among males, 134 (12.7%) received high levels of exposure to the intervention, 408 (38.6%) received moderate levels of exposure, and 515 (48.7%) received no or low levels of exposure.

We re-evaluated the impact of the intervention based on the level of exposure to the intervention the participants received. Given the small numbers of participants receiving a high level of exposure, we combined the moderate and high exposure levels into one group for females during analyses to increase precision in estimating parameters for the fitted analytical models. The results involving four-levels of intervention (control vs. low vs. moderate vs. high) for females can be found in our working paper [23]. Additionally, 67 control participants attended a trial school without peer educators. Since only a small number of control participants received limited intervention exposure, we kept the control group unchanged. We note that our definition of the level of intervention exposure is similar to what was used in the original study but with the additional incorporation of the duration of participants in the community.

**Fig 1:**
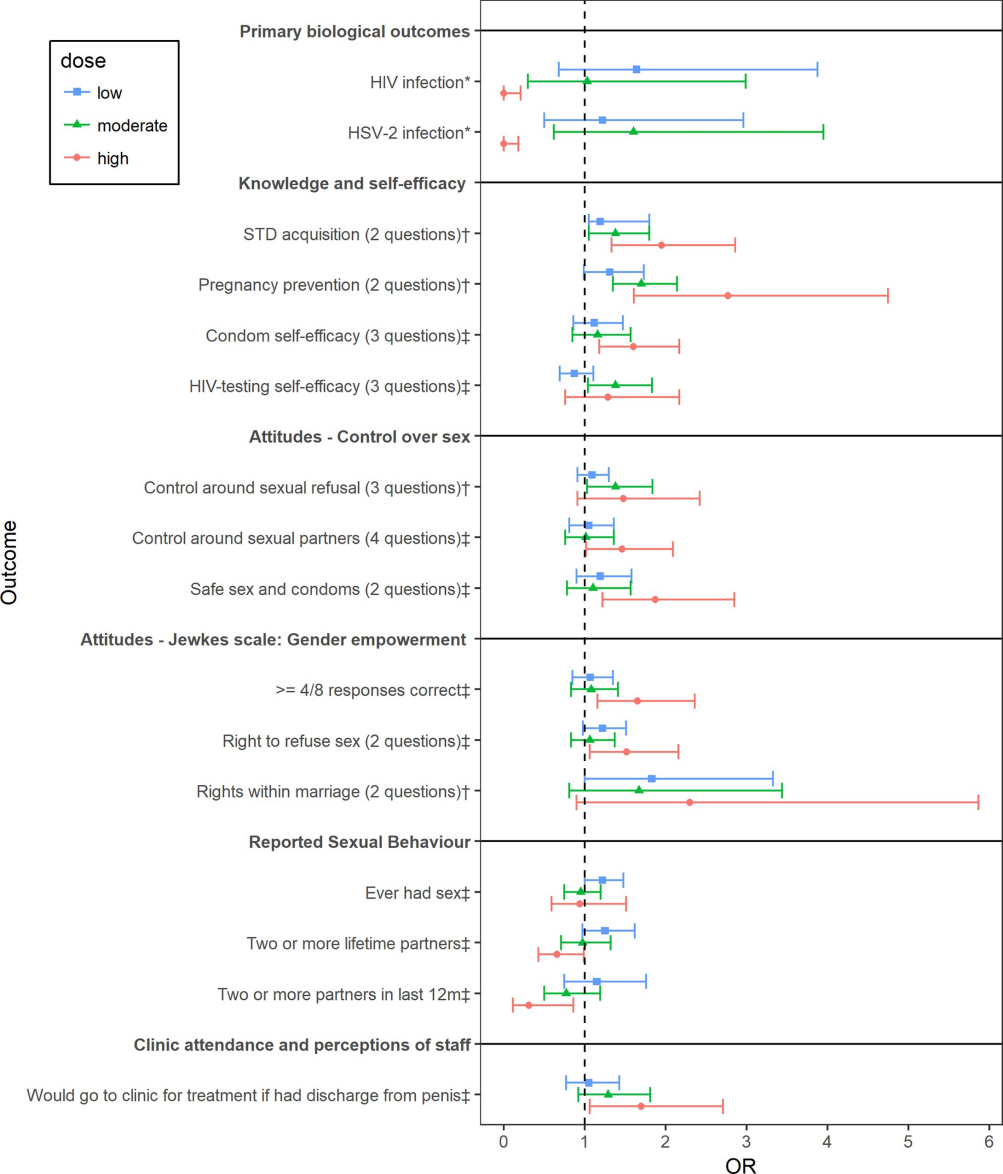
Forest plot showing effects of the intervention at different exposure levels (with reference to control) among males. GEE were used to model on all outcomes, except for HIV and HISV-2 prevalence, which were modelled by Bayesian logistic regression as indicated using *. For the analyses related to “reported sexual behavior”, the reference category includes not reporting the characteristics. The analyses related to “perceptions of staff” were restricted to those who visited the clinic in the last 12 months. AOR and 95% CI estimated by GEE or AOR and 95% credible interval by Bayesian logistic regression with adjustment for age, strata, marital status, and education were displayed. The outcomes that were significantly associated with intervention in the original analyses are identified using †. The outcomes that were significantly associated with intervention only in the new analyses are identified using ‡.

**Fig 2:**
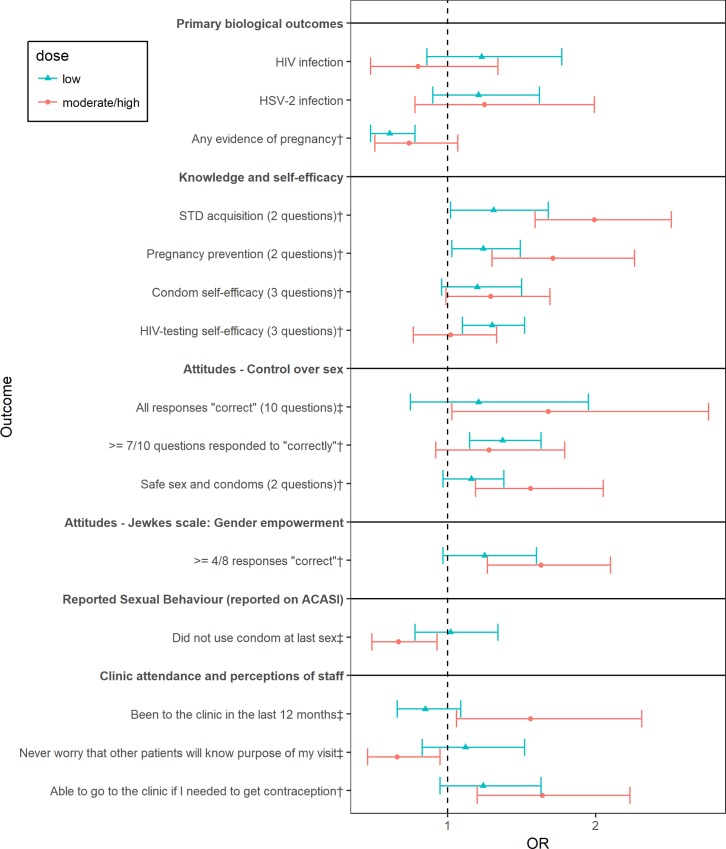
Forest plot showing effects of the intervention at different exposure levels (with reference to control) among females. GEE were used to model on all outcomes. For the analyses related to “reported sexual behavior”, the reference category includes not reporting the characteristics. The analyses related to “reported pregnancy prevention” were restricted to those who reported ever having had sex. The analyses related to “perceptions of staff” were restricted to those who visited the clinic in the last 12 months. AOR and 95% CI with adjustment for age, strata, marital status, and education were displayed. The outcomes that were significantly associated with intervention in the original analyses are identified using †. The outcomes that were significantly associated with intervention only in the new analyses are identified using ‡.

We compared the new findings with the original results by Cowan’s group. The knowledge, attitude, and behavior outcomes that are significantly associated with the intervention in the original study or the new analyses were identified for both males and females. The AOR associated with different intervention levels with reference to control for selected key identified outcomes and all biological outcomes were plotted in Figs 1 and 2.

Among males, the new analyses identified more outcomes that were associated with the intervention, as shown in Fig 1. In addition to the four outcomes (i.e. “STD acquisition”, attitudes toward “pregnancy prevention”, “control around sexual refusal” and “rights within marriage”) that were identified to be positively associated with the intervention in the original analyses, the new analyses also identified nine extra non-biological outcomes that were significantly associated with the intervention in the desired direction. Specifically, the moderate level intervention group was associated with higher odds of having “HIV-testing self-efficacy” (AOR = 1.38; 95% CI = (1.04–1.83)). The high level intervention group was associated with higher odds of responding correctly on “condom self-efficacy” (AOR = 1.60; 95% CI = (1.18–2.17)), “control around sexual partners” (AOR = 1.46; 95% CI = (1.02–2.19)), “safe sex and condoms” (AOR = 1.87; 95% CI = (1.22–2.85)), “right to refuse sex” (AOR = 1.52; 95% CI = (1.06–2.16)), and higher odds of having “4/8 responses correct (gender empowerment)” (AOR = 1.65; 95% CI = (1.16–2.36)). The high-level intervention was also associated with lower odds of having reported risky sexual behavior. For example, the high intervention group had 33% lower odds of having “2 or more lifetime partners” and 69% lower odds of having “2 or more partners in last 12 months” when compared to the control. Lastly, the high intervention group had higher odds of saying yes to “Would go to clinic for treatment if had discharge from penis.”

The new analyses also yielded interesting results regarding biological outcomes among males. No participants in the high exposure groups were detected with HIV or HSV-2 (Table 5); however, the results from GEE analyses failed to provide a valid estimate of AOR for the high-exposure group. Therefore, an alternative Bayesian logistic regression model, as described in the method section was fit. The results of this model showed that the high intervention group was associated with very low odds of having HIV or HSV-2 separately among males.

**Table 5:** The prevalence of HIV and HSV2 by levels of intervention exposure and genders.

Gender	Outcome	Control	Low	Moderate	High
Male	N	994	513	408	134
	HIV	13 (1.3%)	12 (2.3%)	6 (1.5%)	0 (0%)
	HSV-2	15 (1.5%)	10 (1.9%)	9 (2.2%)	0 (0%)
Female*	N	1350	925	300	
	HIV	98 (7.3%)	86 (9.3%)	14 (4.7%)	
	HSV-2	132 (9.8%)	117 (12.7%)	28 (9.3%)	
	N	2344	1438	681	161
Combined	HIV	111 (4.7%)	98 (6.8%)	18 (2.6%)	2(1.2%)
	HSV-2	147(6.3%)	127(8.8%)	36(5.3%)	1(0.6%)

*The participants receiving moderate and high exposure to the intervention were combined for females.

Fig 2 shows that a few more outcomes were associated with the intervention among females in the new analyses. The original analyses showed that the intervention was associated with nine outcomes, which were also shown to be significantly associated with the intervention in the new analyses except for “condom self-efficacy.” In the new analyses, the p-value for testing the association between moderate to high intervention exposure and “condom self-efficacy” is 0.06. The new analyses also showed that the moderate to high exposure group was associated with higher odds of having “all responses “correct” regarding control over sex (AOR = 1.68; 95% CI = (1.03–2.76)) and lower odds of “did not use condom at last sex” (AOR = 0.67; 95% CI = (0.49–0.93)). Additionally, the female subjects exposed to moderate to high levels of the intervention were associated with high odds of “been to the clinic in the last 12 months”, and “never worry that other patients will know purpose of my visit.” However, neither the new analyses nor the original analyses yielded a significant association between the intervention and HIV prevalence or HSV-2 prevalence among females.

We also compared the AOR for the different intervention exposure levels to explore whether there is a trend in the association between higher levels of intervention exposure with outcomes. Males showed the expected trend. The more a male was exposed to the intervention, the greater the magnitude of the odds ratio in the expected direction. In detail, among all four levels of intervention exposure, the larger values of odds ratio were obtained in the higher exposure group for all “knowledge and self-efficacy” (14% to 111% larger AORs in the high vs low exposure group) and “attitudes-control over sex” domains (20% to 51% larger AORs in the high vs low exposure group), except for the “HIV-testing self-efficacy” and attitudes toward “safe sex and condoms.” The high exposure group also exhibited 23% to 73% smaller AOR values of having reported risky sexual behaviors, and smaller odds ratio of having HIV than the low exposure group, as expected.

Females showed more varied results among examined outcomes. A higher level of intervention was associated with greater positive effects on knowledge outcomes, “condom self-efficacy”, and some attitude outcomes. For “did not use condom at last sex,” a higher exposure level resulted in a lower odds ratio, which is the desired effect. Also, the moderate or high level of intervention was associated with less HIV infection when compared to the control, although the results were not statistically significant (AOR = 0.80; 95% CI = (0.48–1.34)). However, an increase in exposure level did not necessarily lead to the desired effect for all outcomes. For example, a higher level of intervention exposure was associated with reduced positive effects on “sexual refusal self-efficacy,” “HIV testing self-efficacy,” “≥ 7/10 correct (attitudes),” “control around sexual partners,” and “long-range goals.” The higher level of intervention exposure was also associated with a larger odds ratio of reporting “ever had sex.”

For the treatment on the treated analysis, Murphy’s score test results indicated excessive kurtosis or skewness in the error distributions, and the model assumptions of the bivariate probit models were violated. Therefore, we omitted these results in this paper and refer readers to our working paper for detailed results [23].

#### MEA for identifying patient subgroups with heterogeneous intervention effects

An individual’s past sexual history did not influence the effects of the intervention regardless of gender or outcome (not reported). However, an individual’s age resulted in different levels of association between the intervention and selected outcomes, as shown in Table 6. Specifically, for males, association of the intervention varied across age groups for the outcomes of “knowledge on STD acquisition” (p = 0.01), “attitudes ≥ 7/10 questions correct for control over sex” (p = 0.03), and “did not use condom at last sex” (p = 0.02). For females, the outcomes were “knowledge on pregnancy prevention” (p<0.01), “HIV-testing self-efficacy” (p = 0.04), and “Jewkes ≥ 4/8 responses correct” (p = 0.05).

**Table 6:** Stratified analysis by age for selected outcomes.

Gender	Endpoint	18 years (N = 752)		19–20 years (N = 711)		21–22 years (N = 616)		Original
		AOR [95% CI] ^1^^,^ ^2^	P-value	AOR [95% CI] ^1^^,^ ^2^	P-value	AOR [95% CI] ^1^^,^ ^2^	P-value	AOR [95% CI] ^1^^,^ ^2^
**Males**	**Knowledge and self-efficacy (% responding “correctly” to questions)**							
	STD acquisition (2 questions)	0.97 [0.70–1.33]	0.84	1.71 [1.24–2.35]	<0.01	1.51[1.30–0.76]	<0.01	1.32 [1.08–1.61]
	**Attitudes–control over sex (% responding “correctly” to questions)**							
	≥ 7/10 questions responded to “correctly”^3^	1.11 [0.79–1.56]	0.55	0.98 [0.72–1.33]	0.91	1.64 [1.21–2.24]	<0.01	1.18 [0.94–1.48]
	**Reported sexual behavior (reported on ACASI)**							
	Did not use condom at last sex^4^	0.97 [0.53–1.78]	0.91	1.57 [1.10–2.24]	0.01	0.73 [0.52–1.03]	0.07	1.03 [0.83–1.29]
**Females**	**Knowledge and self-efficacy (% responding “correctly” to questions)**							
	Pregnancy prevention (2 questions)	1.63 [1.33–2.01]	<0.01	1.02 [0.81–1.28]	0.85	1.53 [1.15–2.03]	<0.01	1.32 [1.14–1.55]
	HIV-testing self-efficacy (3 questions)	1.22 [0.92–1.62]	0.16	0.95 [0.70–1.28]	0.74	1.66 [1.27–2.17]	<0.01	1.22 [1.03–1.44]
	**Attitudes–Jewkes scale: gender empowerment (% responding “correctly” to questions)**							
	≥ 4/8 responses “correct” ^3^	1.03 [0.74–1.44]	0.85	1.32 [0.94–1.84]	0.10	1.29 [1.03–1.61]	0.03	1.32 [1.05–1.66]

Notes

^1^ GEE with an exchangeable covariance structure and robust standard errors adjusted for *a priori* confounders (strata, marital status and education).

^2^ The reference group is the control arm.

^3^ Cut-off set at the median number of “correct” responses.

^4^ The reference category includes not reporting the characteristics and does not exclude those who have never had sex.

For the outcomes associated with significant age and intervention interactions, we performed stratified analyses by both age group and gender. For both genders, the oldest age group (21–22 years) tended to have improved outcomes compared to the non-stratified analysis. For example, the AOR of not using a condom at last sex for the intervention group versus control was 0.73 (95% CI = (0.52–1.03)) among 21-22-year-old males, as opposed to the AOR of 1.03 (95% CI = (0.83–1.29)) in a non-stratified analysis. An interaction was also notable for females regarding their knowledge outcome on pregnancy prevention. The AOR for 21–22 year old females was 1.53 (95% CI = (1.15–2.03)) compared to the non-stratified analysis AOR = 1.32 (95% CI = (1.14–1.55)).

### Results of TOC analyses on the association between knowledge/attitudes and HIV or HSV-2 prevalence

For both ivprobit regressions among males and females separately, the Wald test of exogeneity yielded non-significant p-values, implying that the knowledge, self-efficacy, or attitudes domains were not statistically endogenous [23]. Additionally, the first-stage F-statistics were all smaller than 10. Therefore, the instrument had weak explanatory power for the knowledge and attitudes domains for both genders. Since the knowledge, self-efficacy, or attitudes domains were not endogenous, and the instrument in many cases was weak, which could bias the results, we proceeded using GEEs for our analysis.

An increase in knowledge or attitudes did not have a significant impact on the prevalence of HIV or prevalence of HSV-2, except for the self-efficacy domain when examining HSV-2. The AOR of having HSV-2 associated with one unit increase in the self-efficacy among females was 1.12, with 95% CI (1.00–1.25), which was counter-intuitive. When using factor analysis to combine knowledge and attitudes into one variable, we found no significant association between improvement in knowledge or attitudes with HIV or HSV-2. S2 Table displays the full results of the GEE analyses.

## Discussion

Our replication analysis had two main overarching purposes with regard to Prevention Science: to rule out chance findings [24] and to demonstrate that results obtained in Cowan et al. [2] are robust to variations in the study population and intervention implementation-related factors as well as analytical factors.

The original researchers clearly described their analyses in their paper and provided the necessary documentation to conduct this replication study. Using the original paper as a guide, and the shared data, we reproduced the original results with only minor discrepancies. However, our extended replication analyses led to findings with implications and utility for advising future implementation and further study into strategies related to HIV prevention among youth such as the need for: a) the design, monitoring, and evaluation of the level of implementation of the key interventions, tracking of changes in the study population, and the impact of these on the study population risk profiles and outcomes; b) the design and evaluation of future interventions for specific subgroups of the target youth population based on our additional findings on key outcomes; c) evaluating hypothesized pathways regarding interventions in general and considering additional interventions that could address bottlenecks by pursuing other critical pathways.

The original paper indicated the participants’ migration as a challenge during study implementation. Our analyses revealed these as non-trivial regarding their impact on the findings. Specifically, we identified study subjects who lived in the community for less than five years as newcomers. We found that newcomers were quite prominent in the study population, with about one-quarter of males and nearly one-half of females being newcomers to the study population following the baseline study.

In describing the limitations of their study, the original authors indicated a high possibility that the population was different between the baseline and final evaluation time in ways that may have affected their findings, but there is no detailed description of this observation and its impact on the results. In our analysis, we compared the subject characteristics between the newcomers and others who lived in the community for longer than five years. Our results show that the study population changed in five key characteristics. Newcomers were older, more educated, more likely to include married females and not reporting economic difficulties. Conversely, among the intervention group, newcomers were less likely to have attended an intervention school. Age, education, marital status, and economic status in the direction observed are generally accepted as mitigating risk related to sexual behavior associated with HIV and STDs in sub-Saharan Africa. The introduction of newcomers with a lower risk profile likely led to fewer sexual behavior outcome events and therefore, lower power to show an impact on the key sexual behavior and biological outcomes of interest.

We also revealed the impact of a significant reduction in the level of exposure to the envisaged intervention on outcomes. Although the original authors noted inadequate levels of exposure to the intervention in the study population, our analyses indicate that this was more severe than was noted after we incorporated an assessment of not only the direct exposure to the intervention but also the duration of participants in the community during the study. We incorporated the duration in the community as it may affect the level of exposure achieved by the participants. Only 2% of females and 13% of males were fully exposed to the intervention, and three-quarters of females and almost half the males received little or no exposure to the intervention. This meager level of exposure is probably important in explaining the lackluster impact of the intervention.

Our dose-response analysis based on the received exposure level of the intervention showed the critical role of a high level of exposure to the intervention and its impact on key high-risk sexual behaviors related to biological outcomes, which is different from the original results. Specifically, we revealed that the intervention had a significant effect on sexual behavior outcomes, including a lowering of odds of having multiple partners [25] for males with high exposure to the intervention, and an increase in odds of using condoms during sex for females with high exposure to the intervention. While the original authors noted no effect of the intervention on any aspect of clinic attendance, we revealed that among those with high exposure, female subjects were less likely to worry about perceptions related to purposes of their clinic visits and were significantly more likely to have attended a clinic in the prior year. Similarly, male subjects with high exposure to the intervention were significantly more likely to attend a clinic if they had discharge from the penis. These results are noteworthy since key pillars of the strategy to address the HIV epidemic among adolescent and young women 15–24 years old and their partners as highlighted in the ABC and more recent comprehensive strategies include the reduction of partners, use of condoms, and the need to promote access to comprehensive structural services. Also, while the original study showed no impact on biological outcomes, there is an indication from our Bayesian analyses of a significant reduction of HIV and HSV-2 prevalence among male subjects who had high exposure and a trend towards a decrease in risk for HIV infection among similarly exposed female subjects.

Additionally, the more a participant was exposed to the intervention, the greater the magnitude of the odds ratio of the outcome in the expected direction when compared to a control. Males consistently exhibited this trend, while females deviated from this trend for selected outcomes. These differing trends among groups of participants could be a result of an unmeasured heterogeneity in the risk profiles of those with varying levels of intervention exposure, some of which is hinted at in our analyses by the disparity between males and females in the proportion of newcomers who have different risk profiles.

It is critical that those involved in conducting similar pre-post studies and program evaluations try to assess the participant and community resident losses and gains, and address how the study population change affects the effective evaluation of key outcome indicators and/or exposure to all key aspects of the planned intervention. The strategy of addressing and or mitigating these issues should ideally be made a priori during study or program design, or subsequently via appropriate analyses that take into consideration the received level of exposure or the characteristics of the study populations generated by changes in population. It is also essential that studies of this kind quantify or at least elaborately describe how any significant losses/gains in key participant sub-groups may have affected intervention exposure, outcome evaluation, and interpretation of findings.

The quality of implementation of preventive interventions when delivered in “real-world” settings is often suboptimal, and implementation fidelity is known to influence intervention outcomes [26]. Assessing implementation fidelity and quality of implementation, including documentation of modifications that occur in the field during scale-up, should be a key activity at all stages of any study during program implementation [27–28]. It enables us to understand the extent to which the core components of the intervention can be varied based on aspects that relate to levels of exposure observed while still achieving the desired effect. It also helps us understand whether any potential lack of effect is due to a failure in implementation or of the intervention. It is essential that such information is collected, assessed, and reported in all trials. This information can then provide a benchmark against which later trials or intervention scale-up can be compared. Therefore, we believe a clear and complete description of the intervention is necessary, especially in instances such as this study, where changes occurred during the study, to guide practice, provide a basis for sound measurement of its implementation, and for replication.

High exposure to this study intervention seems to result in a reduction in some key risk behaviors related to the ABC strategy. We propose for a more diligent and formal design, monitoring and evaluating aspects of the study that relate to the level of exposure, and identifying fundamental changes that may have occurred in the study population itself. Research that separately achieves optimal exposure of this study intervention as described by the high exposure group in our analyses is still necessary to confirm the effectiveness in the reduction of key risk behaviors and is worth pursuing.

Our study demonstrated the importance of designing and evaluating an intervention in a way that allows for disaggregation by specific subgroups of the target population for HIV prevention. Our analyses yielded various results associated with outcomes and exposure based on age and gender, highlighting the importance of pre-specifying and analyzing for heterogeneity and interaction effects across key subgroups such as age and gender in the assessment of evidence from confirmatory trials. It also indicates that even in this narrow age spectrum (ages 15–24), youth cannot be treated as homogenous in developing an intervention. Therefore, we recommend key subgroup evaluation in similar multi-component interventions that may have a varied impact on specified subgroups of the target population.

Our TOC analysis shows how the intervention may influence HIV and HSV-2 outcomes, and help new intervention developers or those that may need to adapt the current intervention to identify features of the intervention that are most central to the action theory. Chen [29] and MacKinnon [30] indicate two components of the theoretical mechanism regarding how an intervention affects key outcomes: the “action theory”, which corresponds to how the intervention will affect mediators, and the “conceptual theory”, which focuses on how the mediators are related to the outcome variables. Our replication TOC analysis provides an assessment of original study intervention based on these two theories. A possible pathway, based on these theories, is that the original intervention may decrease HIV or HSV-2 prevalence by improving the knowledge of sexual health and/or attitudes toward sexual risks. The original study evaluated the intervention effects on reducing HIV or HSV-2 directly. They also showed, like we did, that the intervention had an impact on knowledge. In our TOC analysis, we examined whether an increase in knowledge or attitudes or combined would be sufficient to decrease HIV or HSV-2 prevalence. We found no statistically significant results in the desired direction. Knowledge and HIV risk reduction skills-building have previously been linked to behavioral change [31–34]. Such HIV/AIDS prevention is based on the belief that increasing an individual’s knowledge will motivate participants to alter the behaviors that put them at risk for contracting HIV [35]. However, as shown in our results, recent studies in sub-Saharan Africa have indicated that knowledge, for most adolescents, especially those in rural settings, is not consistent with expected risk reduction outcomes [36]. On the other hand, our observation of a more robust impact of the intervention on risky sexual behavior and biological outcomes in males suggests that together with the intervention incorporated in this study, further interventions that address the root distal causes of risky sexual behaviors, such as socio-economic inequalities like poverty and gender disparities, especially among rural female adolescents, may be more effective for HIV risk reduction.

## Conclusion

The considered behavior intervention had an effect on improving knowledge and attitudes. As the level of exposure increased, the intervention showed higher effects on improving knowledge and attitudes, clinical access, and reducing some risky sexual behaviors. The intervention also showed a trend towards reducing HIV or HSV-2 prevalence among males with a high-level exposure to the intervention.

For this study intervention to have the desired effect regarding key sexual behavior and perhaps biological outcomes, it is vital that it incorporates implementation strategies that lead to the achievement of consistently high exposure targeted particularly at males. More research is needed to design an intervention that maximizes the amount of exposure to the intervention that various subgroups of participants receive in the intervention arm.

The examined intervention incorporated improvement in knowledge, attitudes, and sexual behaviors similar to key components of the HIV/AIDS ABC strategy. The results of this study provide further support of these intervention components as key parts of a comprehensive public health strategy for preventing HIV and STDs. However, it appears that a focus on knowledge and attitudes alone, especially among females, may not be sufficient to impact key risky sexual behavior and/or reduce HIV and HSV-2 prevalence.

Additional and/or complementary interventions that act outside knowledge and attitudes-based pathways perhaps with more direct effect in reducing risky sexual behaviors and improving the use of key services, via changing social norms, incentivizing risk reduction behavior, improving the ability to negotiate past common barriers, should also be investigated [37]. For example, such additional interventions may focus on skills-building and breaking down structural bottlenecks to access as well as promoting empowerment towards the consistent and proper use of condoms, better recognition of STD symptoms, more effective health-seeking behaviors, postponing sexual debut, and reducing the number of partners.

There isn’t unilateral support of the impact of the intervention for both males and females on several key outcomes, particularly for females. Therefore, females possibly have a greater need for other complementary intervention strategies that address empowerment and gender disparities.

Finally, we believe that, using methodology similar to that of our replication, a formative evaluation of the action and causal theory of components of an intervention, an assessment of a given intervention’s usefulness at varied levels of exposure, and an evaluation of impact to be expected for key subgroups of the target population should be standard practice and would better advise the use and scale-up of the evaluated interventions in various contexts.

## Supporting information

S1 Table Intervention impact on knowledge, self-efficacy and attitudes outcomes analyzed using ordinal regression. (DOCX)

S2 Table GEE regression on the association between knowledge and attitudes domain with HIV prevalence or HSV-2 prevalence. (DOCX)
