# Supplementary material for: Preventing HIV and HSV-2 through knowledge and attitudes: A replication study of a multi-component community-based intervention in Zimbabwe
Source: PLoS One. 2020 Jan 8;15(1):e0226237. doi: 10.1371/journal.pone.0226237 (PMC6949002; doi:10.1371/journal.pone.0226237)
Supplement: S1 Table — (DOCX) [file pone.0226237.s001.docx]

**S1 Table: Intervention impact on knowledge, self-efficacy and attitudes outcomes analyzed using ordinal regression.** All outcomes were categorized into four categories depending on their quartiles. Ordinal logistic regressions with standard errors allowing for intragroup correlation and adjusted for *a priori* confounders (age, strata, marital status, and education) were fitted on all outcomes (with reference to lower quartiles) among males or females, except for modelling knowledge on HSV, STD and pregnancy prevention (indicated using 1) among females, which were modelled using multinomial logistic regression with the first quartile as reference. The 25^th^, 50^th^, 75^th^ and 100^th^ quartiles for answering seven questions on knowledge of HIV, STD and pregnancy prevention were 2, 4, 6, 7 for males and 2, 4, 5, 7 for females. The 25^th^, 50^th^, 75^th^ and 100^th^ quartiles for answering eight questions on self-efficacy regarding condom use, sex refuse and testing were 5, 6, 7, 8 for both males and females.

The 25^th^, 50^th^, 75^th^ and 100^th^ quartiles for answering ten questions on attitudes toward control over sex were 5, 7, 8, 10 for males and for females. The 25^th^, 50^th^, 75^th^ and 100^th^ quartiles for answering eight questions on attitudes toward Jewkes scales were 2, 4, 5, 7 for males and 2, 3, 4, 7 for females.

|  | **Males** | **Females** |
| --- | --- | --- |
|  | AOR [95% CI] | AOR [95% CI] |
| **Knowledge and self-efficacy** | | |
| HIV/STD & Preg. Prev. (7 ques.) ^2^ | 1.49 [1.26-1.77] | 1.64 [1.30-2.08]^1^ |
| Condom, sex refuse, testing (8 ques.) ^3^ | 1.06 [0.89-1.28] | 1.29 [1.08-1.54] |
| **Attitudes – Control over sex** | | |
| Sexual refuse & safe sex (10 ques.) ^4^ | 1.20 [1.00-1.43] | 1.32 [1.10-1.58] |
| **Attitudes – Jewkes scale: Gender empowerment** | | |
| Jewkes (8 ques.) ^5^ | 1.27 [1.07-1.51] | 1.28 [1.05-1.55] |
