# Supplementary material for: Preventing HIV and HSV-2 through knowledge and attitudes: A replication study of a multi-component community-based intervention in Zimbabwe
Source: PLoS One. 2020 Jan 8;15(1):e0226237. doi: 10.1371/journal.pone.0226237 (PMC6949002; doi:10.1371/journal.pone.0226237)
Supplement: S2 Table — (DOCX) [file pone.0226237.s002.docx]

### **S2 Table: GEE regression on the association between knowledge and attitudes domain with HIV prevalence or HSV-2 prevalence.** Intervention effects were estimated using GEE with adjustment for priori confounders (age, strata, marital status & education), an exchangeable covariance structure and robust standard errors. Adjusted odds ratio and 95% confidence interval for a 1 unit increase in the specified domain were reported.

|  |  | **Males** | | **Females** | |
| --- | --- | --- | --- | --- | --- |
| **Endpoint** |  | **AOR [95% CI]** | **P Value** | **AOR [95% CI]** | **P Value** |
| **HIV** |  |  |  |  |  |
|  | Knowledge (7 questions) | 0.95 [0.80-1.12] | 0.55 | 1.02 [0.94-1.10] | 0.60 |
|  | Self-Efficacy (8 questions) | 0.98 [0.82-1.17] | 0.83 | 1.06 [0.95-1.18] | 0.32 |
|  | Attitudes (9 questions) | 1.01 [0.79-1.30] | 0.92 | 1.07 [0.98-1.16] | 0.12 |
|  | Jewkes (4 questions) | 1.21 [0.83-1.75] | 0.32 | 1.01 [0.84-1.22] | 0.91 |
|  | Factor Analysis | 0.95 [0.72-1.26] | 0.74 | 1.05 [0.90-1.22] | 0.51 |
| **HSV-2** |  |  |  |  |  |
|  | Knowledge (7 questions) | 1.06 [0.88-1.28] | 0.52 | 1.03 [0.96-1.11] | 0.36 |
|  | Self-Efficacy (8 questions) | 1.06 [0.89-1.26] | 0.53 | 1.12 [1.00-1.25] | 0.049 |
|  | Attitudes (9 questions) | 0.96 [0.80-1.14] | 0.61 | 1.01 [0.93-1.10] | 0.74 |
|  | Jewkes (4 questions) | 0.94 [0.66-1.33] | 0.73 | 1.03 [0.89-1.19] | 0.70 |
|  | Factor Analysis | 1.03 [0.78-1.35] | 0.84 | 1.13 [0.99-1.30] | 0.07 |
